# Blockchain-Based Access Control and Behavior Regulation System for IoT

**DOI:** 10.3390/s22218339

**Published:** 2022-10-30

**Authors:** Haoxiang Song, Zhe Tu, Yajuan Qin

**Affiliations:** 1 School of Electronic and Information Engineering, Beijing Jiaotong University, Beijing 100044, China; 2 National Engineering Research Center of Advanced Network Technologies, Beijing 100044, China

**Keywords:** blockchain, Internet of things, access control, smart contract, locator/ID separation protocol

## Abstract

With the development of 5G and the Internet of things (IoT), the multi-domain access of massive devices brings serious data security and privacy issues. At the same time, most access systems lack the ability to identify network attacks and cannot adopt dynamic and timely defenses against various security threats. To this end, we propose a blockchain-based access control and behavior regulation system for IoT. Relying on the attribute-based access control model, this system deploys smart contracts on the blockchain to achieve distributed and fine-grained access control and ensures that the identity and authority of access users can be trusted. At the same time, an inter-domain communication mechanism is designed based on the locator/identifier separation protocol and ensures the traffic of access users are authorized. A feedback module that combines traffic detection and credit evaluation is proposed, ensuring real-time detection and fast, proactive responses against malicious behavior. Ultimately, all modules are linked together through workflows to form an integrated security model. Experiments and analysis show that the system can effectively provide comprehensive security protection in IoT scenarios.

## 1. Introduction

In recent years, with the development and commercialization of 5G, an increasing number of devices are interconnected through wireless networks, forming the Internet of things (IoT). IoT is a promising technique [1]; it provides meaningful information to us and brings convenience to our day-to-day life [2]. However, it can also raise serious cyber-security [3] and data privacy issues. For example, home cameras allow people to remotely view the status of their homes, but once the devices are illegally hacked, there is potential for the leakage of a large amount of user privacy.

Access control is an information security technology that explicitly regulates and restricts access requesters’ rights through the identity information of visitors and security policy provided within the system [4]; it is an important means of securing data resources. However, traditional access control methods, including mandatory access control (MAC) [5], role-based access control (RBAC) [6], and attribute-based access control (ABAC) [7], are all centralized architectures, which need to rely on a central entity for decision-making. These methods face problems such as single point of failure and judgement transparency, which are difficult to apply to IoT scenarios with large device bases, wide distribution, and diverse network forms.

To solve the above problems, distributed access control architectures have been widely studied, such as the application of distributed databases to access control systems. However, at a time when security issues are increasingly emphasized, an increasing number of scholars believe that blockchain is the best solution. Blockchain originated from the concept of Bitcoin, proposed by Satoshi Nakamoto [8], and has recently evolved to the 3.0 era [9]. It has natural advantages in IoT data security: (1) Blockchain synchronizes ledgers among nodes through a P2P network and consensus mechanism, which is very suitable for geographically distributed IoT devices and can effectively solve the problem of single node downtime. (2) Based on blockchain smart contracts, we can realize automated access control for IoT data resources without manual involvement, saving human resources. (3) The results of contract execution are stored in blocks in the form of transactions, which are easy to audit and trace. (4) A benefit from the application of cryptography is that all transactions on the blockchain cannot be tampered with or deleted, so storing data and executing contracts on the chain are very safe and trustworthy. Therefore, we can combine blockchain and traditional access control models to enable an access control system for IoT.

However, it is not enough to focus on whether a user owns authority to access resources, which is just one aspect of data security in IoT; user behavior is essential.

Here, we define user behavior as normal communication behavior that accesses resources in accordance with legal processes and malicious communication behavior for the purpose of attacking resources or stealing data; behavior regulation refers to the timely detection and stopping of someone’s malicious communication behavior. Currently, most studies focus on whether users can access resources, but neglect the question of what users will do with the resources once they gain access authority, resulting in access systems that lack the ability to identify various network attacks and the dynamic and timely defense methods to deal with them. This leads to the formation of a mutually fragmented pattern of access security and communication security.

Access of massive devices will further enhance security threats; traditional access systems not only need to update their architecture and methods to meet IoT requirements, but also urgently need to implement the detection and regulation of user behavior to provide comprehensive security protection for resources.

To address the above issues, we must integrate multiple security technologies to build a trusted access system. The main contributions of this paper are as follows:

(1) We propose a distributed IoT security system architecture that uses the advantages of blockchain. The intelligent gateway entity is introduced between users and IoT resources for building and maintaining the blockchain, and security functions such as access control are implemented based on the blockchain, which can effectively reduce the burden of lightweight IoT devices to perform the above tasks and ensure the security of data resources.

(2) A smart contract-based access control approach is proposed. Smart contracts are designed and deployed on the blockchain to automatically execute processes of authentication and authorization, achieving a fast, flexible, dynamic, fine-grained, distributed, and transparent access control, which solves the difficulty of applying traditional access control methods to the IoT.

(3) A user behavior regulation method is proposed. Traffic detection methods are deployed at every smart gateway to monitor user communication traffic in real time; a credit evaluation method is designed for assessing historical user behavior in the system, and an inter-domain communication mechanism is designed based on the locator/identifier separation protocol (LISP) [10] to block malicious users’ communication behavior at the egress of access network in time.

(4) All methods that apply security technologies, such as authentication, access control, and traffic detection, are fully integrated through a workflow, forming a trusted security model of “user identity—access control—communication behavior—security feedback”, which can proactively detect network threats and quickly respond. The security of IoT data resources is fully guaranteed.

The rest of the paper is organized as follows: in Section 2 we discuss related studies. Section 3 highlights the architecture and framework of our proposed system. Section 4 introduces the functions of each module in the system framework in detail. In Section 5, we describe the system’s workflow. Section 6 summarizes the functional and performance analysis of the system by experiments. In Section 7, we focus on comparison with other schemes and Section 8 summarizes the research content of this paper and provides an outlook on future research directions.

## 2. Related Work

Many solutions have been proposed in the IoT scenario to solve the security issues of data resources by exploiting different techniques closely related to our approach.

### 2.1. Traditional Access Control Method

With the popularity of IoT in daily life, data security and privacy issues are becoming increasingly important, and researchers have proposed various access control schemes to secure safe access to data resources.

In [11], Uddin et al. considered that existing access control models lack dynamic segregation of duties (SoD), so they combined RBAC and TBAC to innovatively define an access control approach based on authorized workflow task roles. Simulation experiments showed that this proposed approach could make decisions based on both tasks and roles with a latency of less than 0.25 s, which was commercially viable. In [12], Rao et al. creatively introduced RBAC to multi-user PEKS, where any group of users can connect to the server to search and access encrypted files based on their roles. The proposed approach could protect IoT data resources from both authority and confidentiality perspectives, and experiments showed that role-based PEKS improved efficiency by 90% compared with ordinary multi-user PEKS. In [13], Liu et al. considered the assignment of user roles in manufacturing IoT (MIoT), extracted the role set, policy set, and permission set into directed graphs, and used intelligent planning theory to model, optimize, and solve the authorization problem to ensure fast decision-making and secure access of RBAC in a multi-domain collaboration MIoT environment. In [14], Tuncay et al. considered the problem of embedded web browsers in apps having unconstrained access to program code and user data. They designed a declarative policy language and constructed a fine-grained access control framework called Draco to restrict the access of embedded webs. Experimental results showed that the proposed approach did not require any modification to the Android OS and the overhead of Draco was negligible. However, client programs are often heterogeneous, and solving the problem at the network level will be more widely applicable.

### 2.2. Blockchain-Based Access Control Method

Considering the lightweight and low-power characteristics of most IoT devices, processes such as policy storage, cryptographic operation, or intelligent planning involved in the above schemes need to be executed by a central entity on their behalf, which introduces additional security risks, such as a single point of failure or data tampering. As a result, many scholars have introduced blockchain technology as a distributed trusted entity involved in access control.

The studies from [15,16,17] all regarded the blockchain as a decentralized third party, and implemented role-based access control by storing user roles, permissions, and other information in the blockchain ledger and deploying smart contracts on the blockchain to perform automated authorization processes. Among them, the novelty of [15] was that the authors additionally designed a smart contract for recording the misbehavior of users and this proposed method allowed admin to impose appropriate penalties on users according to the frequency of misbehavior. The advantage of [16] was its use of challenge-response protocol to verify the declaration and ownership of roles, which could improve the security of cross-organizational RBAC, based on blockchain. The contributions of [17] were that the authors integrated re-encryption technology to complete the privacy protection of user role information on the chain and examined the time and space performance of the scheme.

Besides RBAC, ABAC has recently become more popular among researchers. With the development of 5G, users and resources are constantly moving, environment features are changing in real-time, and static access control methods such as MAC and RBAC are facing increasing difficulty in meeting the demand; meanwhile, the massive access in IoT will make it more difficult for coarse-grained access control models such as RBAC to accurately describe access control entities. As a result, ABAC stands out as a dynamic and fine-grained security technology that allows resource owners to define, add, or update policies based on the actual situation, allows admin to define attributes or features of entities from multiple perspectives, and makes authorizations based on integrated consideration of subject, object, and environment attributes. For example, the key ideas in references [18,19,20,21,22] are all about publishing and storing ABAC attributes and policies in the blockchain, invoking blockchain smart contracts to implement access authorization, and conducting experiments to prove the validity and reliability of blockchain-based ABAC. Specifically, ref. [18] featured the use of a Bloom filter in conjunction with smart contracts to improve the query efficiency of ABAC attributes and policies. The unique contribution of [19] was the proposed indirect access model for resources, i.e., the data resources of IoT devices were sent to the server in advance, where the user was required to perform the ABAC process to obtain the resource URL. The contribution of [20] was introducing grouping policy retrieval algorithms to improve the retrieval efficiency of ABAC policies, thus increasing the throughput of the blockchain system. The innovation of [21] was in dividing IoT data into two types, public and private, and designing different storage and access models for them, respectively. The most valuable work in [22] was the simulation of an attacker tampering with blocks through Matlab, to verify the security of the blockchain-based implementation of ABAC.

### 2.3. Other Security Methods

There are many other forms of data resource security protection techniques in IoT, such as firewall, authentication, encryption, intrusion detection system (IDS), etc. In [12], Rao et al. used PEKS and AES to encrypt the data resources of the device. In [17], Wang et al. used cryptography to ensure the privacy of data on the chain. In [22], Liu et al. used PKI to authenticate the identity of access requestors and resource managers. In [23], Mrabet et al. designed an IoT security framework that contained both authentication, firewall, and IDS, but did not explain how the components worked together.

In general, current research on IoT security issues is not comprehensive, and various security technologies are disconnected from each other, meaning that researchers can only continuously patch the system and passively deal with various security issues. We believe that all security technologies are complementary to one other. Authentication technology is used to ensure credible user identity, access control technology is used to ensure credible access behavior, anomaly detection technology is used to ensure credible communication traffic, and only the organic combination of these technologies can constitute a reliable system that provides all-round protection of IoT device resources. This is the current research gap. We propose a blockchain-based access control and behavior control system for IoT, and build a model link relationship of “user identity—access control—communication behavior—security feedback” through authentication, authorization, and traffic detection technologies to turn passive defense into active interception.

## 3. System Overview

### 3.1. System Architecture

**Figure 1:**
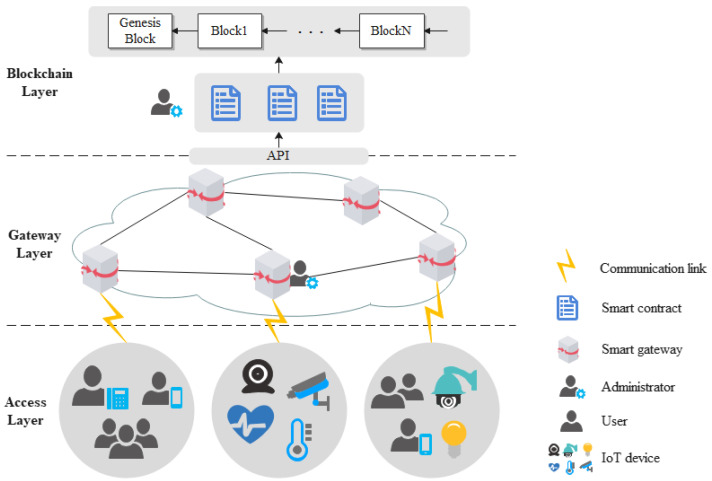
System architecture.

To better solve the problems above, this paper proposes a blockchain-based access control and behavior regulation system architecture, as shown in Figure 1.

- Access Layer

There are multiple independent access domains in the access layer, each of which has a large number of users and IoT devices accessing the system through smart gateways. Users are resource requestors; any user who has an IP address and network communication capabilities can enjoy the services (registration, authentication, access control, and so on) provided by smart gateways and blockchain, but only users authorized by the system are allowed to access the resources of a specified IoT device. Administrators are a special class of users responsible for managing the blockchain, as well as the smart gateways, able to check the blockchain ledger, deploy, update, and delete smart contracts, and verify information of each entity.

IoT devices are responsible for collecting, processing, and sharing data to authorized users. As most IoT devices have limited performance, they are not directly deployed as blockchain nodes; instead, smart gateways act as intermediaries to interact with the blockchain to execute all kinds of processes, such as user information registration, access control policy deployment, and so on.

- Gateway Layer

Smart gateways (SG) are the key to the system’s operation and undertake three main functions. First, each smart gateway manages an access network. Inter-domain communication traffic in the access network first need to reach the smart gateway to be forwarded to the destination through the core network formed by all the smart gateways connected together. Second, a smart gateway acts as a proxy between devices and the blockchain, receiving device requests, invoking the smart contracts deployed on the blockchain for processing, and avoiding the performance and security issues that would be caused by massive devices directly accessing the blockchain. Finally, to reduce the interaction latency with the blockchain, each smart gateway is directly deployed as a blockchain node, and all smart gateways jointly maintain the data on the chain.

- Blockchain Layer

A blockchain is essentially a decentralized database, where data such as entity attributes, access control policies, user access behavior records, and smart contract codes, which will be introduced later, are stored in blocks. All blocks are jointly maintained by all blockchain nodes and are available for administrators to check and trace. Smart contracts are predefined instructions deployed on the blockchain to automatically execute contracts through code programs. As long as the contract terms are met, transactions are automatically carried out without third-party supervision [15]. This layer provides an application interface (API) for smart gateways to call smart contracts and the function modules of the system will fully use these contracts to interact with the blockchain to achieve various business logics.

### 3.2. System Framework

**Figure 2:**
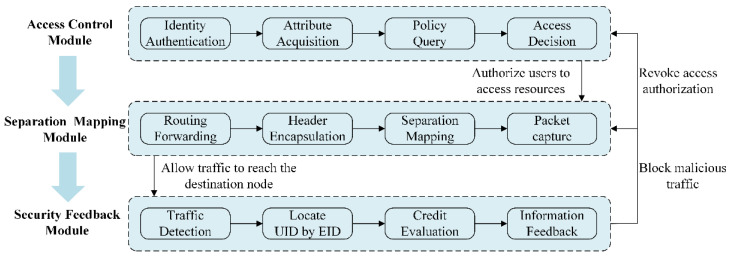
System framework.

To ensure the access and communication security of IoT device resources, we deployed a functional framework at all smart gateway nodes. As shown in Figure 2, this framework provides three important security function modules: the access control module, separation mapping module, and security feedback module.

The access control module is responsible for making access decisions for users who request access to resources and has to ensure the trustworthiness of user identity and decision results by designing a reasonable access control method. The separation mapping module needs to build a new communication mechanism to intercept cross-domain communication packets between users and IoT devices at the egress of the access network, and only packets from authorized users are allowed to reach the destination nodes through the core network, to guarantee that all users accessing the IoT device resources are legal and authorized. The role of the security feedback module is to detect malicious users from the network traffic and update this information to other modules, so that they can respond in time to block malicious users from attacking the IoT devices.

The three modules work together to form a layer-by-layer security protection system that provides access control and behavior regulation to users.

## 4. Module Design

This section provides a detailed description of the three function modules in the framework of a blockchain-based IoT access control and behavior regulation system.

### 4.1. Access Control Module

The access control module mainly performs authentication and access control decision-making processes, and is the first line of defense for the system.

Due to the limited computing abilities of most IoT devices, the identity authentication link adopted a relatively simple account-password authentication method. That is, the user or resource would generate a self-defined key (K) to be saved in the blockchain at the registration stage. Then, during the authentication phase, the user or resource would need to send another K and the blockchain would compare it with the K of registration. If the two keys were equal, authentication would be successful; otherwise, authentication would fail.

Considering the fine-grained requirements of IoT, the access control link adopted the ABAC model.

To describe the characteristics of users and IoT devices in detail, some parameters are defined in this module, as shown in Table 1. Based on these parameters, the access control smart contract (AC_SC) was designed to automatically execute the module function on the blockchain to ensure the transparency and trustworthiness of the execution process.

Before introducing the algorithm designed in this paper, several blockchain built-in functions need to be introduced, because it is with the help of the following functions that we can use smart contracts to perform operations on the blockchain’s data.

- PutState

PutState(a, b) is a built-in function provided by the blockchain that takes two arguments a and b. Argument a should be string type and b can be any data type, such as struct UI or RI in Table 1.

**Figure 3:**
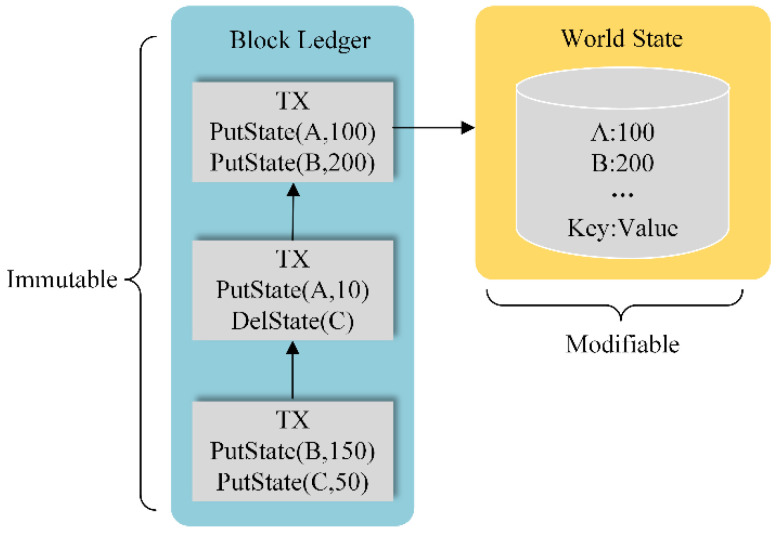
Ledger and database in blockchain.

When we call the function PutState(‘x’, 100) on a smart contract, the blockchain will generate a transaction with the content “set the value of ‘x’ to 100” and write it to the block ledger after consensus. At the same time, the value (or state) of “x” will be updated to 100 in the world state database. The world state database is a non-relational database that stores data through key-value pairs. In fact, most blockchains (including Ethereum and Fabric) maintain two sets of data: one is the block ledger and the other is the world state database, as shown in Figure 3. The block ledger sequentially records the insert, update, and delete operations for all data on the blockchain, which is used for auditing and traceability; whereas the world state database records the newest state of all data in the blockchain, which is used to improve retrieval efficiency.

- GetState

GetState(a) is a built-in function provided by the blockchain that takes one argument. When we call the function GetState(“x”) on a smart contract, the blockchain will query the world state database and return the newest value of “x”. It is to be noted that the process of querying data will not be written into the block ledger, as it does not involve modification of data.

- DelState

DelState(a) is a built-in function provided by the blockchain that takes one argument. When we call the function DelState(“x”) on a smart contract, the blockchain will generate a transaction with the content “delete ‘x’ from ledger” and write it to the block ledger after consensus. At the same time, the key-value pair data ‘x’:100 will be removed from the world state database. Next time we call GetState(“x”), it will return the null.

Based on the parameters and built-in functions above, the algorithms in the AC_SC are described below.

- UID_Register

When the smart gateway receives a user UID registration request, it will invoke Algorithm 1 with input parameters: user-defined UID, K, and user UA, signed by the administrator.
**Algorithm 1** UID_Register**Input**    UID registration information: UID, K, UA**Output**    Registration result1.  result = false;2.  UI = getState(UID);3   check = checkSignature(UA)3.  **if** (UI == null and check == true){4.     new(TS);5.     UI = {UID, K, UA, 0, 0, 0, 0, TS}6.     putState(UID, UI);}7.  **return** true;

Algorithm 1 first checks if the UID has been registered by another user, and subsequently verifies the administrator’s signature on the UA. If the UID is not yet registered and the signature is correct, Algorithm 1 will declare a new struct UI, put the user’s UID, K, and UA into the struct, and store the UI into the blockchain by calling the function PutState(UID, UI).

- RID_Register

Similar to Algorithm 1, the smart gateway receives RID registration request messages from IoT devices and invokes Algorithm 2. Algorithm 2 puts the registration information of IoT devices into a new struct RI and stores it on the blockchain by calling function PutState(RID, RI) to implement the IoT device registration and policy deployment.
**Algorithm 2** RID_Register**Input**    RID registration information: RID, K, PS**Output**    Registration result1.  result = false;2.  RI = getState(RID);3.  **if** (RI == null){4.     RI = {RID, K, PS}5.     putState(RID, RI);}6.  **return** true;

- Access_Control

When a user asks for access authorization, the smart gateway will invoke Algorithm 3 to perform the access control process. Algorithm 3 is the core of the access control module.
**Algorithm 3** Access_Control**Input**      Access control information: UID, RID, K, AA, EA, D**Output**      Access control result1.  result = false;2.  RI = getState(RID);3.  UI = getState(UID);4.  **if** (UI == null){5.     **return** false;}6.  **if** (UI.K ! = K){7.     UI.AuthenF++;8.     putState(UID, UI);9.     **return** false;}10.  UI.AuthenS++;11.  **for** P **in** RI.PS{12.     **if** (UI.UA, EA, AA ⊂ P){13.       result = true;14.       **break**;}15.  }16.  **if** (result == false){17.     UI.AbacF++;18.     putState(UID, UI);19.     **return** false;}20.  UI.AbacS++;21.  Time = time.now( );22.  T = {Time, D, RID};23.  UI.TS.append(T);24.  putState(UID, UI);25.  **return** string(true) + json(T);

(1) Identity authentication

In the ABAC model, different identity characteristics determine different rights. Therefore, to ensure that all access authorizations are correctly granted, Algorithm 3 first checks the user’s identity and considers the user successfully authenticated when the input K is exactly the same as the user registration K stored on the blockchain. Then 1 is added to the field AuthenS in the user’s UI. If user authentication fails, Algorithm 3 will add 1 to the field AuthenF in the user’s UI and this authorization request will be directly rejected; no further steps are performed.

Notably, after the smart contract algorithm is executed, all updates or deletions to data, such as AuthenS or AuthenF on the chain, will be immediately written into the block ledger and updated to the world state database.

(2) Attribute acquisition and policy query

After a successful authentication, Algorithm 3 will then get the user’s UA from UI by calling function getState(UID) and the query resource PS from RI by calling function getState(RID). This step is to prepare for the access control process

(3) Access decision

Finally, an access decision needs to be made. Algorithm 3 will traverse all policies in the resource PS until any policy can match with user attribute UA, environment attribute EA, and user access action AA.

A successful match means that the user has the right to access the resource. A T will be generated for the user, which shows the resource the authorization token can be used to access and how long it is valid for. The token will be written to the field T of the user’s UI and stored on the chain through the built-in function PutState(UID, UI). Eventually all blockchain nodes (smart gateways) will be able to query the authorization token.

Similar to the authentication process, the access control result of the user will lead to an update of the field AbacS or AbacF.

### 4.2. Separation Mapping Module

The function of the separation mapping module is to prevent unauthorized user traffic from reaching its destination through the core network, which is the second line of security for the system. For this purpose, two classes of IP addresses were defined and a new inter-domain communication mechanism was designed based on the LISP protocol.

- Endpoint identifier (EID)

EID refers to the IP address used by users and IoT devices in the access network. EID is only involved in intra-domain routing, not inter-domain routing, and there is no routing information of EID in the core network.

- Routing locator (RLOC)

RLOC refers to the IP address used by the core network routing node (smart gateway); the routing table of the core network contains only the routing entries of the RLOC.

- Inter-domain communication mechanisms

(1) EID registration

When a user or IoT device first connects to the network, they need to perform an EID registration process. The smart gateway will generate mapping information (MI) for the user or device, which is used to bind their EID with the RLOC as well as other parameters of the smart gateway, then updates this MI to other blockchain nodes (smart gateways) by calling blockchain function putState(UID/RID, MI). MI is a struct type with four fields: the EID field is used to store registrant’s EID address; the ID field is used to store the identifier of the registrant, i.e., UID or RID; the RLOC field is used to store the RLOC address of smart gateway; the Flag field is used to indicate whether the registrant is a user or an IoT device.

(2) Using EID for communication

When nodes located in different access networks need to communicate, they use their EIDs as the IP address of all the packets they send.

(3) Packet capture

The smart gateway located at the egress of the access network will capture all cross-domain packets. These packets need to finish the separation mapping process to change their IP address from EID type to RLOC type; otherwise, they will not be addressable in the core network and will finally be dropped.

(4) Separation mapping

For the sake of illustration, consider EID1 as the EID address of the IoT device and EID2 as the EID address of the user. For a packet sent by an IoT device, the smart gateway can directly get its mapping information, such as RID and RLOC, by calling function getState(EID1).

For the user, the smart gateway needs to determine whether the user bound to this EID address has the right to access the resource characterized by this RID. For this purpose, the smart gateway queries the user’s MI stored on the blockchain by calling function getState(EID2), then queries the user’s UI by calling function getState(MI.UID), and gets TS in UI. Once the user’s TS is obtained, all T in the TS will be traversed to verify if access authorization exists for the resource characterized by that RID.

If the authorization does not exist or the authorization time has expired, this means the user is illegally accessing the resource and the smart gateway needs to directly discard the packet; otherwise, the packet belongs to legitimate access traffic, and the next process should be performed.

(5) Header encapsulation [24] and routing forwarding

For every legal packet, the smart gateway encapsulates another layer of IP headers for the packet with the RLOCs of its source and destination EIDs, then routes it to the core network. Other smart gateways in the core network will forward the packet according to its outer header.

(6) Header decapsulation

Eventually, the smart gateway at the other end will receive the packet, remove the outer header, and forward the original packet to the receiving end, and this cross-domain communication process is completed.

With the above mechanism, all illegal packets from unauthorized users can be intercepted at the egress of the access network. Among them, the EID registration process and separation mapping process are implemented by the separation mapping smart contract (SM_SC), to ensure the decentralization of data storage and query. This algorithm is shown in Algorithm 4.

- EID_Register

Similar to Algorithm 3, Algorithm 4 first verifies the user’s identity by comparing K. After success of authentication, Algorithm 4 will make a new struct MI, put the EID registration information into it, and then store it on the blockchain by calling function PutState(EID, MI) to realize the function of EID registration.
**Algorithm 4** EID_Register**Input**    EID registration information: Flag, UID/RID, EID, RLOC, K**Output**    Registration result1.  result = false;2.  UI/RI = getState(UID/RID);3.  **if** (UI/RI == null || UI/RI.K ! = K){4.     return result;}5.  MI = [UID/RID, EID, RLOC, Flag];6.  putState(EID, MI);7.  **return** true;

- EID_Mapping

Algorithm 5 locates the identities of the communicating parties based on the EIDs of packet, verifies the user authorization token, then returns the corresponding RLOC address to achieve separation mapping.
**Algorithm 5** EID_Mapping**Input**      EID1, EID2**Output**      Separation map result1.  MI1 = getState(EID1);2.  MI2 = getState(EID2);3.  **if** (MI1 == null or MI2 == null){4.     **return** false;}5.  **if** (MI1.Flag == ‘UID’){6.     MI1, MI2 = MI2, MI1;}7.  RLOC1 = MI1.RLOC;8.  RID = MI1.ID;9.  TS = (getState(MI2.ID)).TS;10.   **for** T **in** TS{11.    **if** (T.Time + T.D > time.now ( ) && T.RID == RID){12.       RLOC2 = MI2.RLOC;13.       **return** RLOC1, RLOC2;}}14.   **return** false;

### 4.3. Security Feedback Module

The security feedback module is deployed after the access control module and separation mapping module, to monitor access traffic and block malicious traffic in time; it is the third line of security for the system. The working mechanism of the module is as follows:

(1) Traffic detection

To monitor user access behavior, traffic detection methods need to be deployed at smart gateways. Traffic detection methods are a class of software that analyzes traffic data and identifies abnormal traffic in the network through port-based, signature-based, machine learning-based, and deep packet inspection-based methods [25]. It should be noted that our system supports the deployment of any traffic detection method as long as the method is able to analyze network traffic in real time and output detection results containing abnormal IP addresses.

For example, in [26], the author built self-generated dataset by simulating traffic attacks on an experimental platform, used it to train neural networks, combined the trained model and statistics detection model to detect network traffic, and reported detection results in real time. This method is well suited for deployment in our system.

(2) Locating UID based on EID

For the output of abnormal traffic information by traffic detection methods, the system has to figure out that which user is using the EID address for communication. Thus, the identity of the abnormal user (UID) is located by calling function getState(EID) to get the corresponding MI, and the abnormal user’s behavior record and other information are easily acquired by calling function getState(MI.ID).

(3) Credit evaluation

Considering that the traffic detection method has a certain false alarm rate, after locating the identity of the sender of the abnormal traffic, it is necessary to make further judgments in conjunction with their behavior records in the system. The behavior records refer to the parameters, AuthenF, AuthenS, AbacF, AbacS, etc. in Table 1, which are recorded and updated by the blockchain every time the user requests to perform an authentication and authorization process. The abnormal user credit is evaluated by Equation (1). If the credit is higher than a certain threshold, the abnormal user is considered to have good access behavior and identified as a normal user. Otherwise, when both the traffic detection method and credit evaluation deems the user’s behavior abnormal, the abnormal user is identified as a malicious user.

credit = 100 − 50 ∗ AuthenF/(AuthenS + AuthenF) − 50 ∗ AbacF/(AbacS + AbacF)(1)

(4) Information feedback

For malicious users, the inter-module interface is used to feedback their information, and other modules respond in time. Specifically, the access control module will delete the identity information of the malicious user and no longer provide access authorization for him, whereas the separation mapping module will delete the mapping information of the malicious user and no longer forward their traffic to the core network. Finally, the proposed system will successfully stop malicious users from attacking IoT devices.

In the introduction above, steps 2–4 are all implemented on the blockchain through the security feedback smart contract (SF_SC), which guarantees the trustworthiness of the verdict, using Algorithm 6.

- User_Judge

Algorithm 6 implements the identity location, credit evaluation, and user classification for abnormal traffic.
**Algorithm 6** User_Judge**Input**     EID of abnormal traffic**Output**     Credit evaluation result1.  result = false;2.  MI = getState(EID);3.  **if** (MI == null or MI.Flag == ‘RID’){4.     **return** result;}5.  UI = getState(MI.ID);6.  credit = credit_evaluate(UI);7.  **if** (credit < 60){8.     **result** = true;}9.  **else** {10.      **return** result;}

- Delete

Algorithm 7 receives malicious user’s EID as input and removes all information about the malicious user from the blockchain in time, thus completing the security feedback and response process.
**Algorithm 7** Delete**Input**    EID of malicious traffic**Output**    None1.  MI = getState(EID);2.  UID = MI.ID;3.  deleteState(EID);4.  deleteState(UID);5.  **return**;

## 5. Workflow

**Figure 4:**
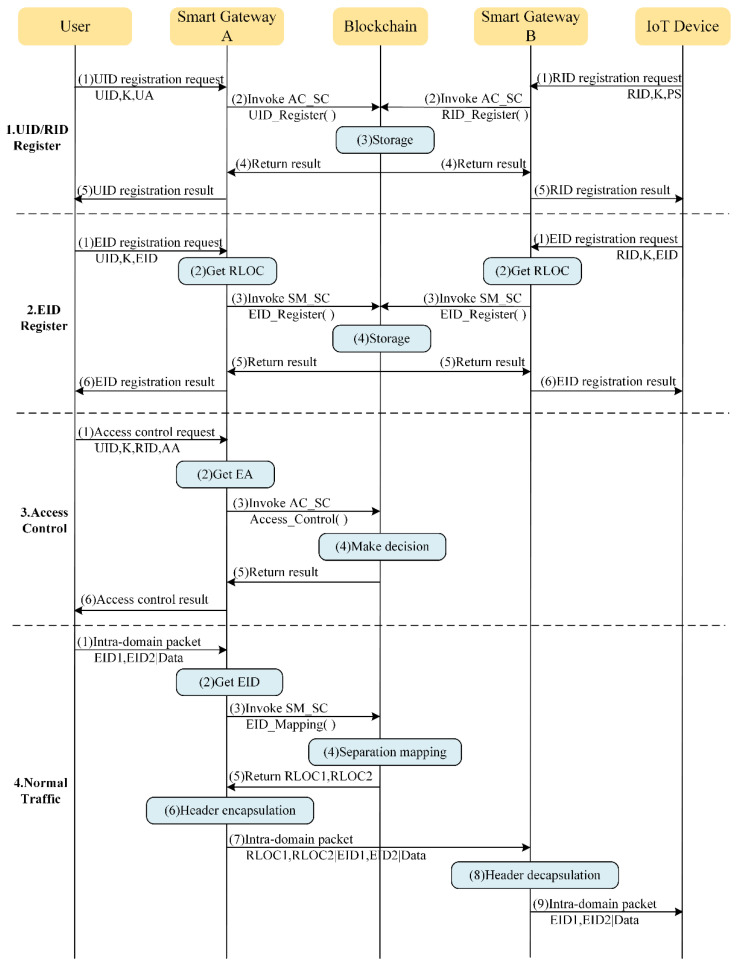
Workflow of system authorizes normal users to access resources.

**Figure 5:**
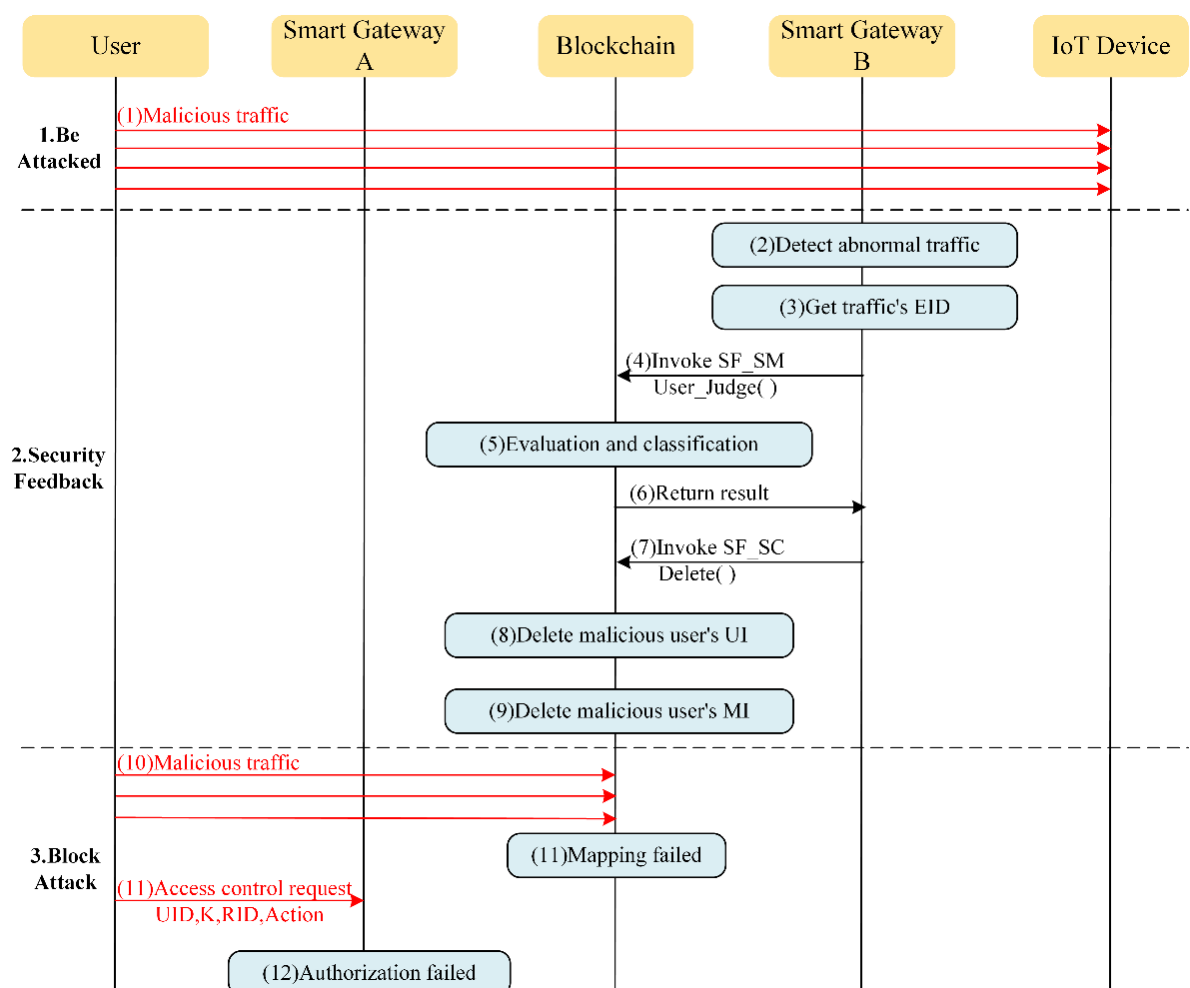
Workflow of system blocks malicious users from attacking resources.

Combining the above three modules, the overall workflow of the blockchain-based IoT access control and behavior regulation system is illustrated in Figure 4 and Figure 5, which introduce the workflow of the system in authorizing normal users to access resources and blocking malicious users from attacking behaviors, respectively. It should be noted that the content privacy of communication process between users or IoT devices and smart gateways are protected by asymmetric encryption algorithms, but they are omitted here for the sake of simplicity.

### 5.1. Normal Access

(1) UID/RID register

When a new user accesses the system, he needs to send a UID registration request to the smart gateway to register information, such as the UID, K, and UA on the blockchain, for the subsequent access control process. 

Similarly, when a new IoT device resource is deployed to the system, the owner of IoT device resource needs to define one or more attribute-based access control policies (PS) based on their own needs and privacy, and sends a RID registration request to the smart gateway to register information, such as RID, K, and PS on the blockchain.

(2) EID register

After completing the UID registration process, users also need to send EID registration requests to the smart gateway to bind their own UID and EID information with the smart gateway’s RLOC, so that the system can find the user’s mapping information and regulate their communication behavior.

IoT device resources that are newly connected to the system also need to send EID registration requests to the smart gateway for binding the RID and EID information with the smart gateway’s RLOC after the process of RID registration.

In addition, whenever a user moves to a new access domain managed by another smart gateway or the user’s EID address has changed, they need to perform the EID registration process again to update their mapping information; otherwise, they will not be able to receive the packets sent to them from others. The situation is the same for IoT device resources, but they are often not mobile.

(3) Access control

In the access control phase, the user sends an access control request to the smart gateway in their net domain, which contains the UID, K, AA, and RID that they wants to access. After the domain smart gateway obtains EA, it calls the Access_Control function of AC_SC to execute the access decision and authorization process, and then returns the result to the user. 

(4) Normal traffic

Once access authorization is obtained, the user can communicate with the specified IoT device for a specified period of time. Both packets between the two can reach the other end through the inter-domain communication mechanism designed by the separate mapping module.

### 5.2. Malicious Attack

(1) Be attacked

As shown in Figure 5, an adversary is launching a malicious traffic attack to an IoT device.

It should be noted that both normal users and adversaries are able to request the smart gateway to perform the UID registration and EID registration process for them. The only difference is that normal users own a UA verified and signed by the administrator whereas adversaries do not. Therefore, without a valid UA, adversaries have difficulty accessing resources. This leads to adversaries attempting to gain access by stealing the passwords of normal users. If an adversary obtains a password, they easily gain access authorization under the identity of normal users, binding the stolen user’s UID with their own EID by updating mapping information, and immediately launching an attack. Hackers these days have greater means of obtaining user keys, such as using brute force cracking, forging phishing scam websites, planting monitoring programs on the user end, using sniffers, etc. Therefore, we believe that no authentication technology is absolutely secure.

Assuming that the adversary is performing a malicious attack of an IoT device under the identity of a normal user who does have the right to access the attacked device, we can observe how our proposed system stops the attack.

(2) Security feedback

As the traffic detection method is deployed, the smart gateway is able to detect anomalies in the bidirectional traffic between the user and the attacked IoT device within a short period of time after the start of the attack.

Subsequently, the smart gateway gets the EID address of the abnormal traffic, calls the User_Judge function of SF_SC to locate the identity of the abnormal user, and calculates the user’s credit. As the adversary illegally stole a normal user’s identity, the values of parameters such as AuthenF and AbacF will be relatively high (normal users know their keys and the devices they have rights to access, whereas the adversary does not, so the adversary sends many access control requests until they crack the key and find a target device that can be accessed and attacked), which leads to the calculated user credit to be lower than the threshold. Thus, the abnormal user would be identified as a malicious user. Next, security feedback would send this information to the other two modules. Once the feedback message is received, the access control and separation mapping modules will respond in time to delete the EID and UID information of the normal user who have had their passwords stolen by the adversary.

(3) Block attack

Thereafter, attack packets from malicious users will be discarded by the smart gateway and access control requests will be denied, thus implementing regulation of user behavior in the real-time detection, response, and blocking of malicious attacks.

In some cases, the adversary may eventually find that the identity information they stole is no longer available and plan to find other users for identity theft and other resources that can be used for attack. However, when most user keys are complex, and the smart gateway sets up a more secure request processing mode (for instance, the user is told to wait ten minutes for three consecutive failed authentications), the cost of time or resources for the attacker to crack the key will be very high. In contrast, the normal user whose identity was stolen by the attacker, they only need to perform UID and EID registration again and appropriately increase the complexity of their key to restore normal access to the system.

The workflow above is the process of the coordinated operation of each module, which can ensure resource security by building connections between “user identity—access control—communication behavior—security feedback”.

## 6. Experiment

To verify the feasibility of our proposed blockchain-based IoT access control and behavior regulation system, a prototype system was built in a realistic environment and several experiments were conducted.

### 6.1. Experiment Environment

**Figure 6:**
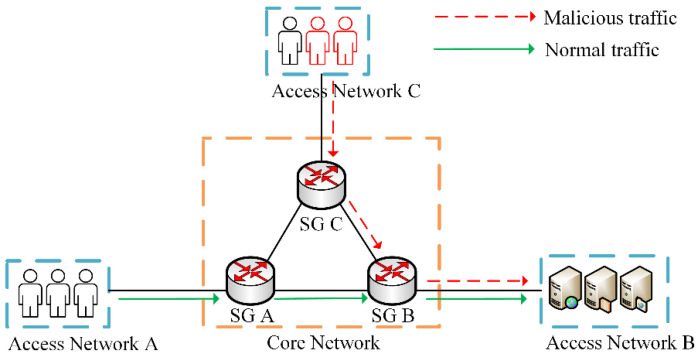
Experimental network topology.

The experiments were conducted on the Vmware vSphere virtual platform, using 9 virtual machines to simulate 3 normal users, 1 malicious user, 2 IoT devices, and 3 smart gateways. The virtual machines had Intel^®^ Xeon^®^ CPU E52609 v4 @1.70 GHz *8, 8 GB RAM, 1 TB HDD, Ubuntu 20.04.1 LTS, Golang version v1.11, Docker version v20.10.7, Hyperledger Fabric version v1.4. 3, Ethereum version v1.10.1-stable-c2d2f4ed. The network topology built by the 9 VMs is shown in Figure 6, and the network configuration information is shown in Table 2.

### 6.2. Blockchain Framework Comparison

From a technical point of view, a blockchain can be divided into a public blockchain and consortium blockchain. To explore which blockchain would be more suitable for the blockchain-based IoT access control and behavior regulation system proposed in this paper, we compared public blockchains and consortium blockchains from both efficiency and security perspectives.

We collected the searched volume of some mainstream blockchain platforms on three search engines, as shown in Table 3. As Bitcoin does not support smart contracts, the most representative blockchain development platforms are currently Ethereum [27] and Fabric [28]. Most scholars also tend to build an Ethereum [15,16,17] or Fabric blockchain [2,18,19,20,21] for experiments. Thus, the public blockchain framework used to perform experiments was Ethereum, which was developed by the Ether Foundation organization; the consortium blockchain framework was Fabric, a project developed by an IBM-led company.

First, the Ethereum blockchain and Fabric blockchain were respectively built on all smart gateways shown in Figure 6, and the access control module was also deployed. Smart contracts on the Ethereum blockchain were written by Solidity and smart contracts on the Fabric blockchain were written by Golang.

**Figure 7:**
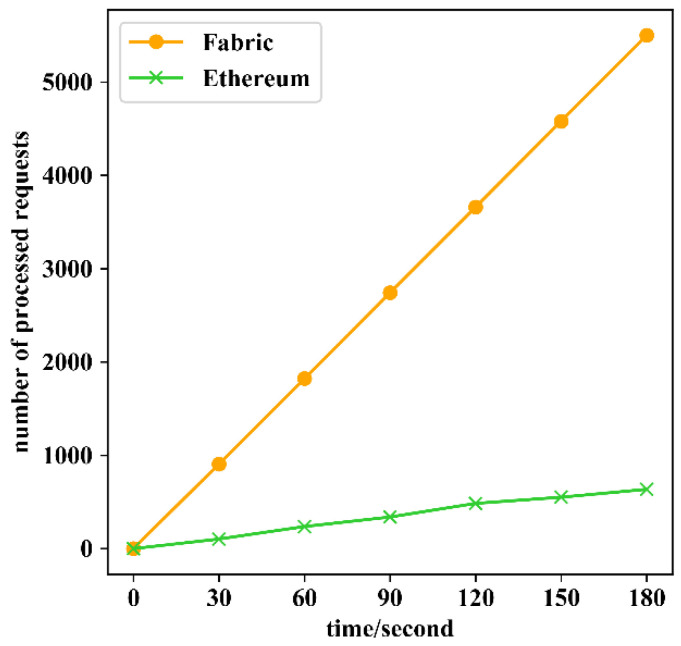
Comparison of the number of access control requests processed.

Subsequently, access control requests were continuously sent from user A1 at a constant rate and the cumulative number of requests processed by SG A was measured, as shown in Figure 7. The experimental results showed that a single smart gateway could process a total of 5501 requests through Fabric in 180 s, with an average of 30.6 requests per second; in comparison, Ethereum could only process a total of 636 requests in 180 s, with an average of 3.53 requests per second. 

**Figure 8:**
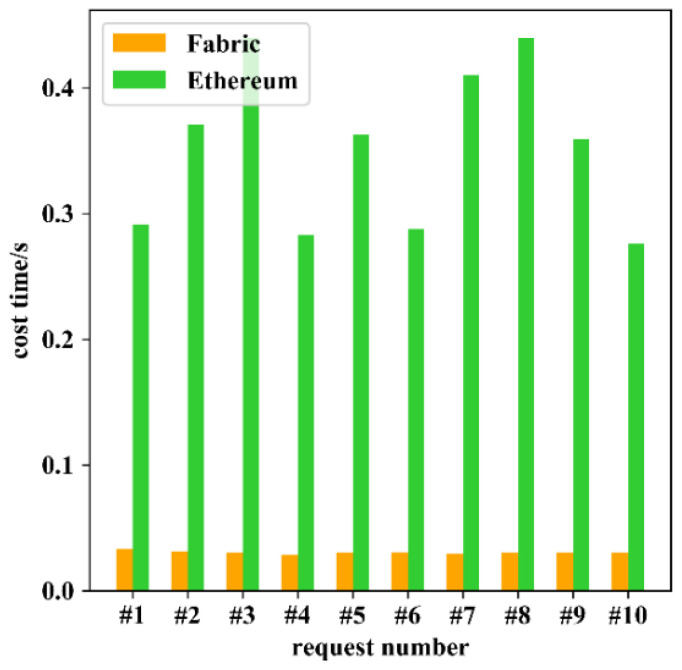
Time cost of processing a single access control request.

Next, the time cost of processing a single access control request was tested. As shown in Figure 8, a single smart gateway took only 0.03 s to process an access control request through Fabric, with less fluctuation; on the other hand, it took 0.68 s on average to process an access control request through Ethereum, which was more than twenty times longer than the former.

These two sets of experimental data showed that execution efficiency of smart contracts was much faster in Fabric than in Ethereum, caused by the different consensus mechanisms of the two. The former uses a simple and efficient consensus RAFT, whereas the latter uses the proof of work (PoW), which consumes more computing power and has lower performance.

In terms of security, a public blockchain does not have any restrictions on participants, so participants usually participate in on-chain activities with anonymous identities and are allowed to access the transactions in the blocks; on the contrary, a consortium blockchain pays more attention to the privacy and security of data on the chain, requires participants to have clear identities, and implements real-name management of all participant identities through certificates issued by authoritative institutions.

The system designed in this paper needed to meet the demand for massive access and to regulate user behavior, while also ensuring the privacy of users and IoT devices, thus a consortium blockchain such as Fabric was a better choice. The subsequent experiments were carried out in the system built on the consortium blockchain, Fabric.

### 6.3. Function Test

To verify the feasibility of the functions of each module, we conducted several experiments.

First, normal user A1 in the access network A sent three access control requests to SG A to apply for access of the IoT device B1.

**Figure 9:**
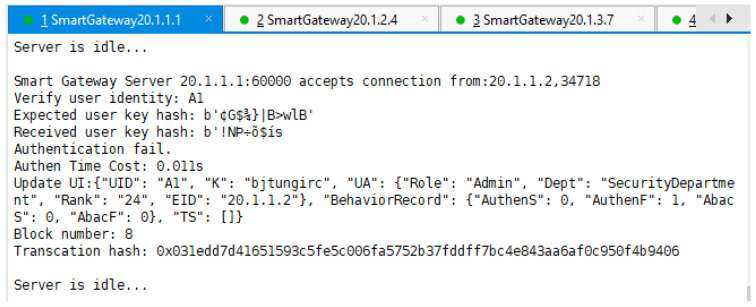
SG A performs access control process and the result is authentication failure.

As shown in Figure 9, as we intentionally entered the wrong key, SG A invoked the contract with the result of authentication failure.

**Figure 10:**
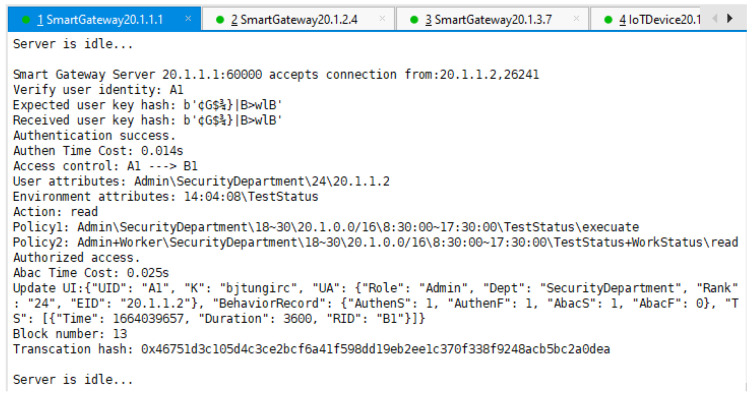
SG A performs access control process and the result is authorization success.

Next, we proceeded to send the same request, but with the correct key. As shown in Figure 10, the result of the contract run shows that A1 successfully matches Policy 2 of B2 and the authorization is successful.

**Figure 11:**
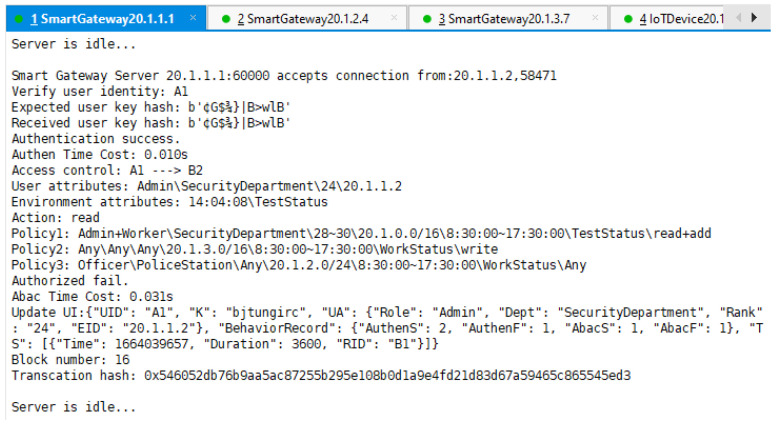
SG A performs access control process and the result is authorization failure.

We also tried to let A1 request access to resource B2. As we can see from the results in Figure 11, A1 did not meet any of the policies deployed by B2, so the result was authorization failure.

From the three figures, we can also see how the fields AuthenS, AuthenF, AbacS, AbacF, and TS in A1’s UI were updated according to different authentication and authorization results.

Meanwhile, malicious user C2 in access network C was running two threads: (1) constantly sending a large number of TCP connection requests to B1 to simulate DoS attacks and (2) making an attempt to obtain C1’s K by means of brute force cracking, obtaining authentication and authorization under the identity of C1, and making the attack traffic avoid being blocked by the separation mapping module.

**Figure 12:**
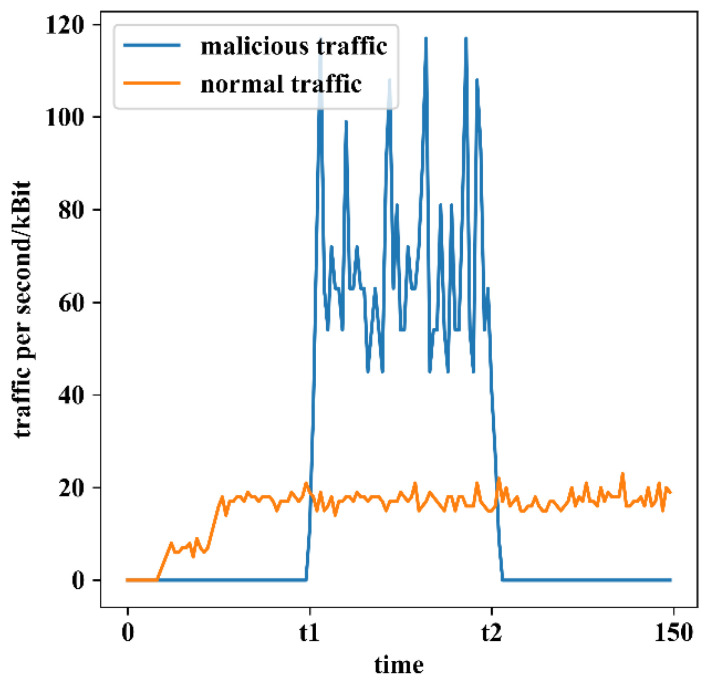
Network traffic statistics at IoT device B1.

Throughout the experimental phase, we measured the traffic statistics of B1, as shown in Figure 12.

**Figure 13:**
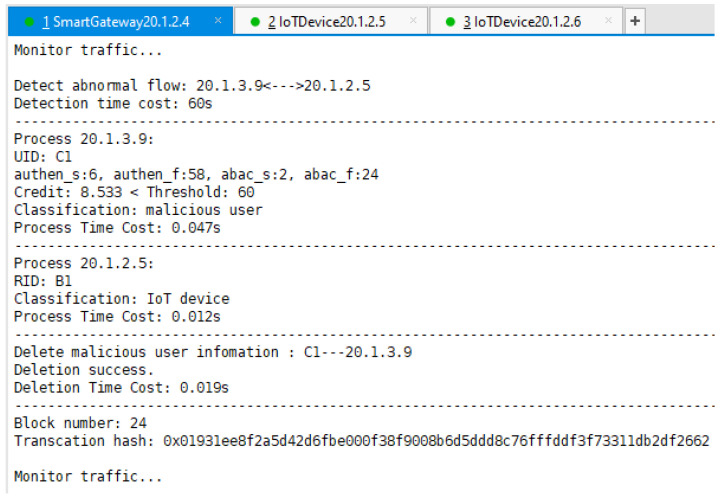
SG B performs the security feedback process.

We see that from time 0 to t1, as malicious user C2 does not gain access authority, the attack traffic cannot reach B1 because the separation mapping module blocks it. During t1 to t2, B1 suffers from a DoS attack because the malicious user C2 successfully steals C1’s identity and obtains the access authorization token at moment t1. However, our system deploys some traffic detection methods [29,30,31,32] designed by other researchers in our research team (ref. [29] for application DDoS, ref. [30] for low-rate DDoS, ref. [31] for Botnet, and ref. [32] for DRDoS). Thus, at moment t2, SG B detects the abnormal traffic produced by C2 and immediately responds through the security feedback module, as shown in Figure 13. Eventually, all packets from C2 with EID address 20.1.3.9 are blocked at SG C, thus protecting the IoT device B1 from C2’s malicious attacks. In comparison, the communication traffic of normal users remain unaffected.

From Figure 13, we can also see that the whole process from B1 being under malicious attack by C2 to the blocking of the malicious traffic took about 61 s, whereas the detection module took 60 s to detect the malicious traffic (60 s is the time window set by reference [29] and time window is a parameter that is highly related to the accuracy of detection), and the process of locating user identity, calculating user credit, reporting malicious user information, and deleting malicious user information took less than a second.

**Figure 14:**
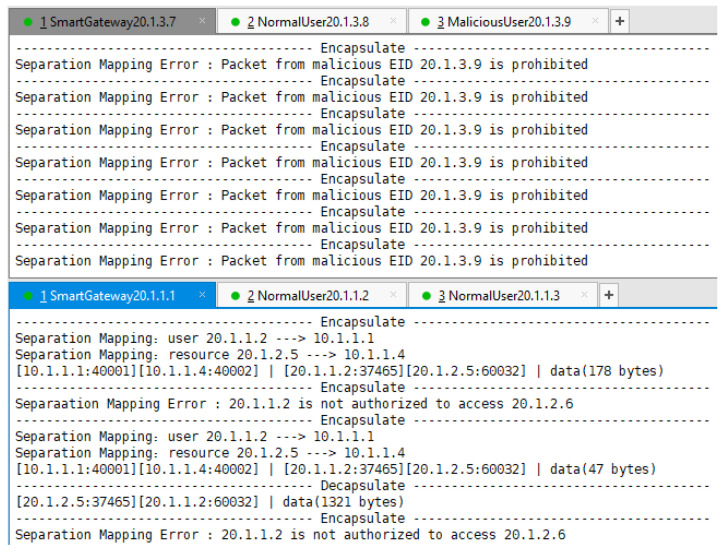
SG C and SG A perform the separation mapping process.

The output of SG’s separation mapping module at runtime in Figure 14 is a good verification of the feedback results. For SG C, it continuously captured C2’s packets but refused to provide separation mapping services for them because the security feedback module was informed that EID 20.1.3.9 was a malicious IP address. For SG A, it provided decapsulation services for the packets flowing into access net A, provided encapsulation services for packets from A1 to B1, but refused to encapsulate packets from A1 to B2 because A1 only had access authorization for B1, which is shown in Figure 9 and Figure 11 above.

In summary, the above experiments illustrate the successful implementation of the functions of the access control module, separation mapping module, and security feedback module.

### 6.4. Performance Evaluation

In the blockchain-based IoT access control and behavior regulation system, processes such as policy query and user information query belong to “read operation”, i.e., reading information from the blockchain; UID, EID, or RID registration belongs to “write operation”, i.e., writing information to the blockchain. The access control process is a combination of read operation, write operation, and the execution of smart contract code to implement ABAC business logic. In the following section, we will evaluate system performance by testing the speed of read and write operations performed in the blockchain.

**Figure 15:**
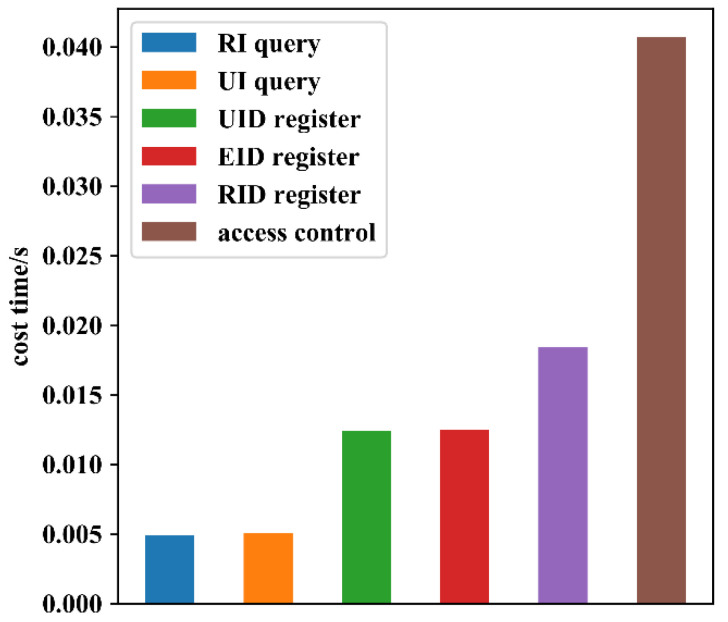
Time cost of a single smart gateway for processing various types of requests.

As shown in Figure 15, the time cost of a single smart gateway for processing various types of requests was tested by simulating the sending of multiple requests through A1. The results showed that the read operation was the fastest, the write operation was slower, and the access control process was the slowest. This is because the writing operation needs to generate blocks, perform node consensus, and execute ledger synchronization, which means it contains more processes than reading blocks. However, in general, the time cost of processing a single request did not exceed 50 ms and the processing rate was acceptable.

**Figure 16:**
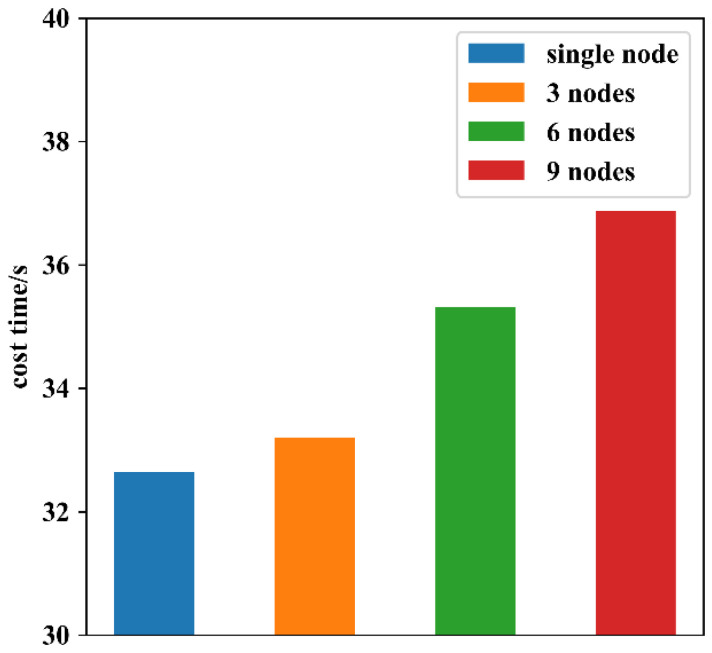
Impact of the number of blockchain nodes on the processing speed of an individual node.

The number of blockchain nodes (i.e., smart gateways in this paper) also had an impact on system performance; thus, eight additional virtual machines were configured to act as blockchain nodes for testing. During the test, 1000 concurrent access control requests were sent to SG A to measure how long it took for a single node to finish a fixed number of requests as the number of blockchain nodes changed. As shown in Figure 16, the processing efficiency of a single node slightly decreases as the number of nodes in the blockchain increases; this is because the increase in the number of nodes slows down the consensus mechanism. Therefore, to achieve a good balance between performance and scale, the administrator must set a suitable number of nodes according to different requirements when applying this system.

**Figure 17:**
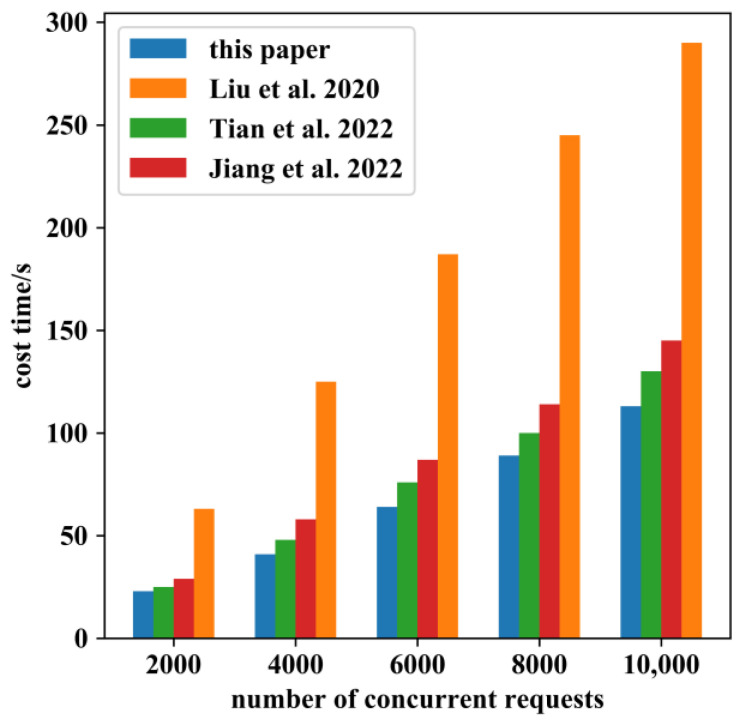
Time cost of access control process at different numbers of concurrent requests which compared with the results of the methods proposed by Liu [19], Tian [20], Jiang [21] respectively.

Subsequently, the processing capability of our system in the face of a large number of burst requests was examined. During the test, nine smart gateways were deployed in the system to process multiple concurrent requests sent by users. The results in Figure 17 show that the system designed in this paper exhibited better performance in processing many concurrent requests compared with other systems or methods [19,20,21]. The reason for this was: (1) the authentication step filtered out some illegal users and saved time in performing subsequent steps; (2) the system had a simple, but reasonable contract design; (3) we chose a consortium blockchain and used consensus algorithms that were efficient (RAFT) or supported high concurrency (Kafka).

**Figure 18:**
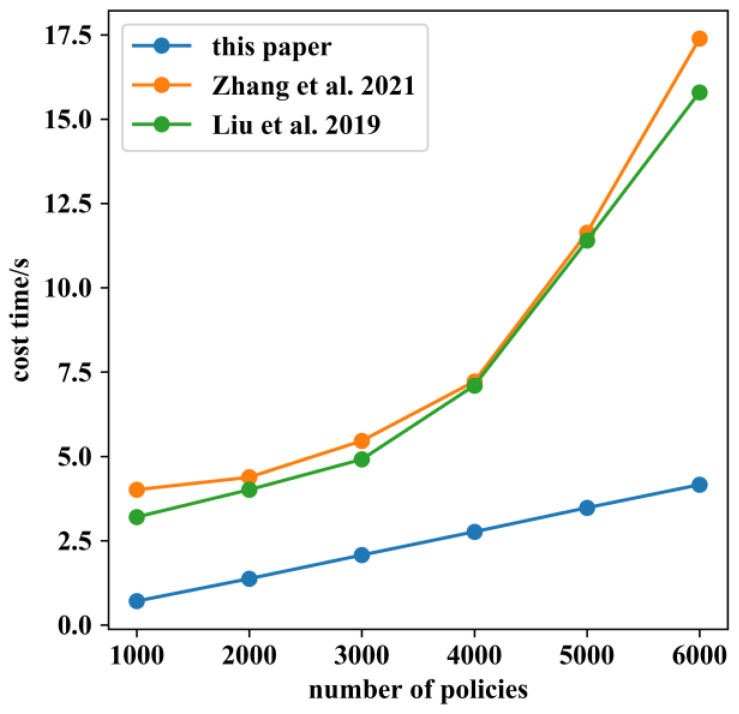
Effect of number of policies on time cost of access control process which compared with the results of the methods proposed by Zhang [18] and Liu [22] respectively.

In the experiments above, we defaulted to deploying only three policies per IoT device. ABAC allows an object to deploy many of policies to meet more fine-grained access control demands. To analyze the impact of the number of policies on access control process, six sizes of policy numbers, 1000, 2000, 3000, 4000, 5000, and 6000, were sequentially deployed on the blockchain through the IoT device B2, then 100 access requests for B2 were sent from user A2 to SG A; the measured time costs are shown in Figure 18. The results showed that the time cost of the access control process was positively related to the number of policies, because all policies need to be traversed for correct authorization results. As our system used the blockchain world state database to query the newest state of policy, which avoided the process of traversing all ledgers, the time cost in this paper was less than [18,22].

## 7. Contrast Analysis

This section evaluates the proposed access control and behavior regulation system by comparing some existing research schemes. The system described in this paper had the advantages shown in Table 4.

- Distributed

Access control business logic was realized through smart contracts deployed on the blockchain. Due to the decentralized technical characteristics of the blockchain, distributed access control decision and authorization were successfully realized, which could solve the problem of single points of failure.

- Fine-grained

Due to the application of the ABAC model, access control entities were described by a variety of attributes, which enabled a more fine-grained division of access control entities. The ABAC model also allows us to deploy more flexible access control policies compared with traditional models, such as RBAC and ACL.

- High security

The Fabric blockchain itself has a certain access mechanism that can protect user privacy; the designed access control mechanism can prevent users from overstepping their rights to access resources; traffic detection methods can monitor user traffic access in real time; the security feedback module can identify malicious users; and the separation mapping module can block malicious traffic.

- Transparent decision-making

User attributes, resource policies, and access control records were stored on the blockchain, where administrators could trace the user access control execution process through blockchain ledger data and logs at any time, which ensured the transparency of decision-making.

- Massive access

The performance evaluation section reflected the advantages of our system in terms of processing speed compared with other studies. A faster processing speed means the system is stronger at processing massive concurrent access requests. The application of LISP made the core network’s routing table contain only RLOC addresses and not the EID addresses of massive access devices. Thus, the core network routing table was highly convergent and scalable, which meant the system could withstand the impact of massive node access on core network routing performance.

- Proactive blocking attacks

The deployment of traffic detection methods and design of the security feedback workflow enabled our system to sense malicious traffic in real time, turning passive defense into active interception.

## 8. Conclusions and Prospects

This paper focused on the security of data resources in IoT. We first introduced a blockchain-based ABAC method to achieve automated authentication and authorization by smart contracts, which solved the problem that traditional access control methods face in meeting the needs of distributed, fine-grained, and tamper-proof security in IoT. Then, in order to implement the regulation of user access behavior, we innovatively introduced the separation mapping module and security feedback module. The former identified malicious traffic based on IDS and a credit evaluation mechanism, whereas the latter filtered malicious traffic in the core network based on LISP, thus ensuring that IoT device resources were protected from a malicious user’s attacks in the process of being accessed. Finally, by designing a complete workflow, we successfully integrated mainstream security technologies, such as authentication, authorization, and IDS, to form a security model of “user identity—access control—communication behavior—security feedback”. Experiments and comparative analysis results showed that our system was functionally complete, had excellent performance, and were actively intercepting attacks in terms of security, which is important for IoT security-related fields.

For future research work and directions, we propose the following prospects:

(1) The throughput of the blockchain should be improved. In the future, we would try to combine verifiable computation (VC) [33] with the blockchain to sink the heavy computational task in blockchain smart contracts to the user side, so that the blockchain only needs to verify the correctness of user computation results through VC, add the correct computation results to the block ledger, and update the corresponding world state database. This would allow the blockchain to implement the maintenance of data on the chain, rather than running contracts, improving its throughput.

(2) The capabilities of smart gateways should be scaled. In this paper, resources and policies were manually configured by IoT device owners. In the future, we could try to let the smart gateway act as a data collector to proactively detect the device resources in the access network, automatically deploy access control policies for each new IoT device, and quickly and safely collect all data resources based on constrained trees [34], giving the system intelligence.

(3) The credit evaluation mechanism could also be perfected. Currently, the threshold value of the credit evaluation mechanism is manually specified by the system administrator, which is not flexible enough. In the future, we would try to train an optimal threshold output model by means of reinforcement learning, etc., so that it could dynamically adjust the threshold value according to the current network environment. This would reduce the probability of a normal user being misclassified as a malicious user, while not missing any malicious users.

(4) More experiments need to be carried out on this topic. Due to limited time and resources, our system faced several limitations, which are listed above. In the future, we propose to equip more physical machines for testing the performance of our system in face of massive requests or huge communication traffic. Also, we intend to test the impact of Fabric blockchain configuration parameters (e.g., maximum number of bytes per block or maximum number of transactions per block) on the system to find the best configuration parameters applicable to our system.

## Figures and Tables

**Table 1:** Description of parameters.

Parameter	Description
User Identity (UID)	String type; used to characterize the user’s identity
IoT Device Identity (RID)	String type; used to characterize IoT device and the data resources it generates
Authentication Key (K)	String type; generated during the registration phase, used to authenticate the user or IoT device
User Attributes (UA)	String type; used to represent user’s characteristics, such as role, age, department, and job
Environment Attributes (EA)	String type; used to represent the environmental characteristics of the system when access control occurs, such as time, temperature, and security level
Access Action (AA)	String type; used to represent user’s operations on IoT device resources, such as read, write, execute, etc.
Access Control Policy (P)	Logical discriminant type; input UA, EA, and AA and output Boolean value; True means the user currently satisfies the policy P
Policy Set (PS)	Array type; all P deployed by the same IoT device resource constitute a PS; if user’s access request satisfies any P of a resource’s PS, the user is considered to have the right to access the resource
IoT Device Information (RI)	Struct type; contains three fields: RID, K, and PS
Authorization Token (T)	Struct type; generated by smart contract and stored on the blockchain when the user successfully passes the access control process, and is a kind of credential that authorizes the user to communicate with the specified resource. It contains three fields: Time, D, and RID. Time indicates the moment when the user finishes the access control process; D indicates the duration for which the system allows user access; RID indicates which resource the token is used to authorize user access
Token Set (TS)	Array type; all T obtained by the same user constitute a TS
AuthenS	Int type; used to record the number of successful authentication attempts for a user
AuthenF	Int type; used to record the number of failed authentication attempts for a user
AbacS	Int type; used to record the number of successful authorization attempts for a user
AbacF	Int type; used to record the number of failed authorization attempts for a user
User Information (UI)	Struct type; contains eight fields: UID, K, UA, AuthenS, AuthenF, AbacS, AbacF, and TS

**Table 2:** Network configuration information.

Network	Network Number	Node	IP Address
Access Network A	20.1.1.0/24	Normal User A1	EID: 20.1.1.2
		Normal User A2	EID: 20.1.1.3
Access Network B	20.1.2.0/24	IoT Device B1	EID: 20.1.2.5
		IoT Device B2	EID: 20.1.2.6
Access Network C	20.1.3.0/24	Normal User C1	EID: 20.1.3.8
		Malicious User C2	EID: 20.1.3.9
Core Network	10.1.1.0/24	SG A	EID: 20.1.1.1 RLOC: 10.1.1.1
		SG B	EID: 20.1.2.4 RLOC: 10.1.1.4
		SG C	EID: 20.1.3.7 RLOC: 10.1.1.7

**Table 3:** Comparison of the searched volume of different blockchain platforms.

Searched Volume	Google Scholar	Baidu	Microsoft Bing
Bitcoin	215,000	58,900,000	113,000,000
Ethereum	66,500	26,800,000	421,000,000
Hyperledger Fabric	23,500	7,960,000	364,000,000
Corda Blockchain	6300	526,000	61,700,000
Hyperchain	456	369,000	41,400

**Table 4:** Comparative analysis of this paper and existing research schemes.

Schemes	Distributed	Blockchain	Model	Fine-Grained	Security	Transparent Decision-Making	Massive Access	Proactive Blocking Attacks
[11]	×	-	TRBAC	×	Access control mechanism, extended XACML policy language	×	×	×
[12]	×	-	RBAC	×	Access control mechanism, multi-user PEKS	×	×	×
[15]	√	Ethereum	RBAC	×	Access control mechanism, reward and punishment mechanism based on access records, blockchain	√	×	×
[17]	√	Ethereum	RBAC	×	Access control mechanism, re-encryption technology, blockchain	√	×	×
[18]	√	Fabric	ABAC	√	Access control mechanism, asymmetric encryption algorithm, blockchain	√	×	×
[19]	√	Fabric	ABAC	√	Access control mechanism, blockchain	√	×	×
[20]	√	Fabric	ABAC	√	Public key infrastructure, blockchain	√	√	×
This paper	√	Fabric	ABAC	√	Blockchain, a security model of “user identity—access control—communication behavior—security feedback”	√	√	√

## Data Availability

The data involved in this paper are available from the corresponding author upon reasonable request.
